# Supplementary material for: Shoot‐through layers in upright proton arcs unlock advantages in plan quality and range verification
Source: Med Phys. 2025 Aug 19;52(9):e18051. doi: 10.1002/mp.18051 (PMC12364776; doi:10.1002/mp.18051)
Supplement: Supplementary file 1 — Supporting information [file MP-52-0-s001.pdf]

Supplementary material

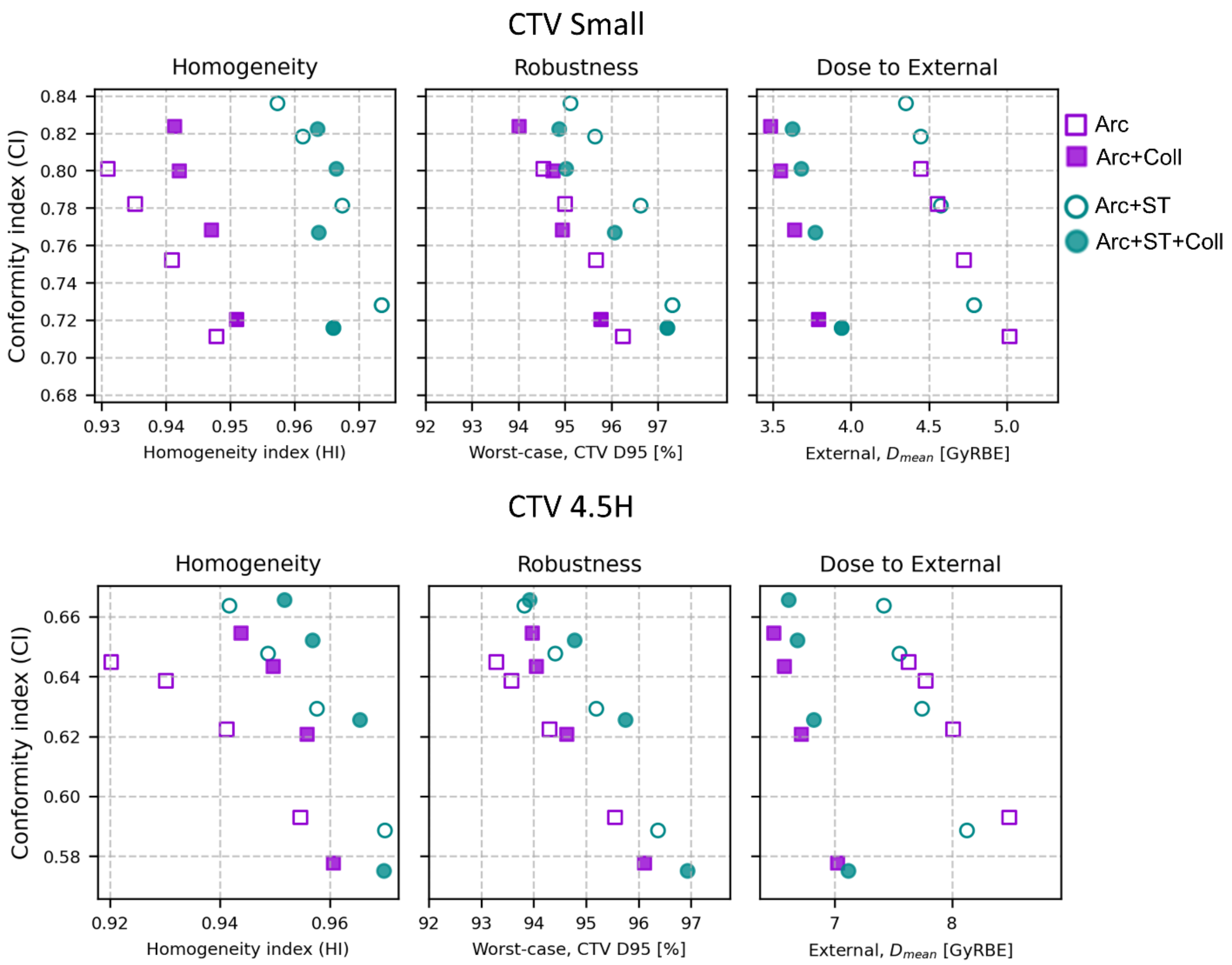

Figure S1. Same evaluation as in Figure 3 but for CTV Small and CTV 4.5H.

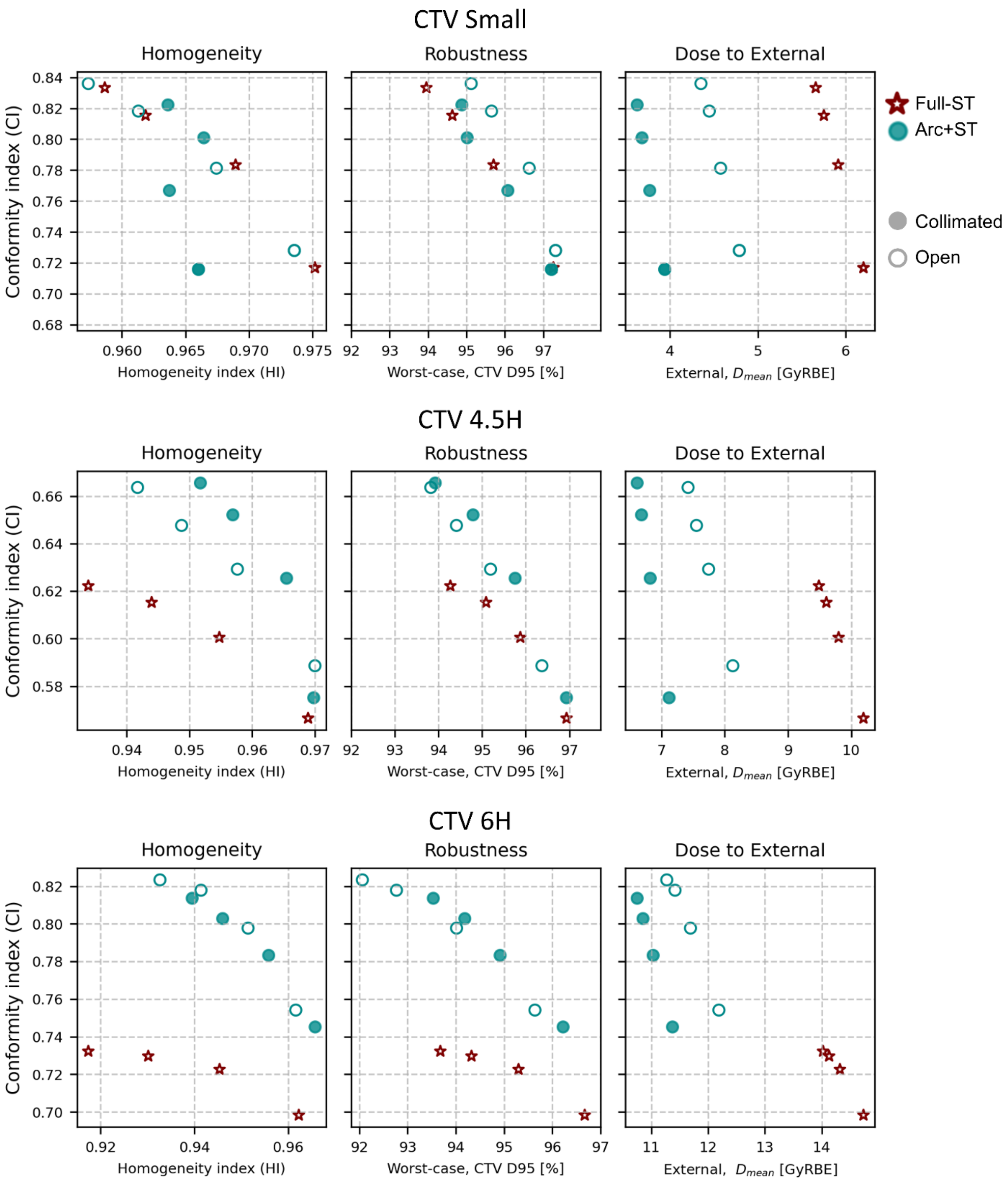

Figure S2. Comparison between Arc+ST and Full-ST for CTV Small, CTV 4.5H and CTV 6H. The Full-ST plans have the same number of directions (20) as the Arc+ST, but only employs a single ST layer from each direction.

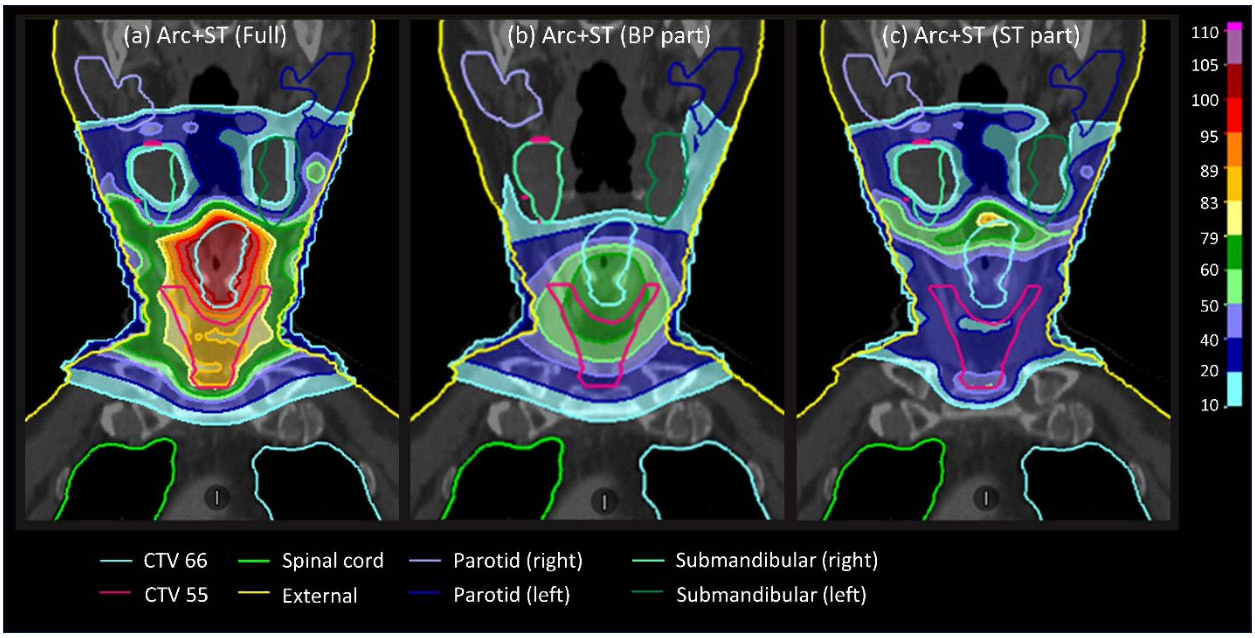

Figure S3. Coronal view of the Arc+ST plan. Panel (a) shows the full plan, whereas panels (b) and (c) show the Bragg peak (BP) and ST parts, respectively. It can be seen that a large portion of the ST protons are placed at the border between the CTV 66 and the submandibular glands.

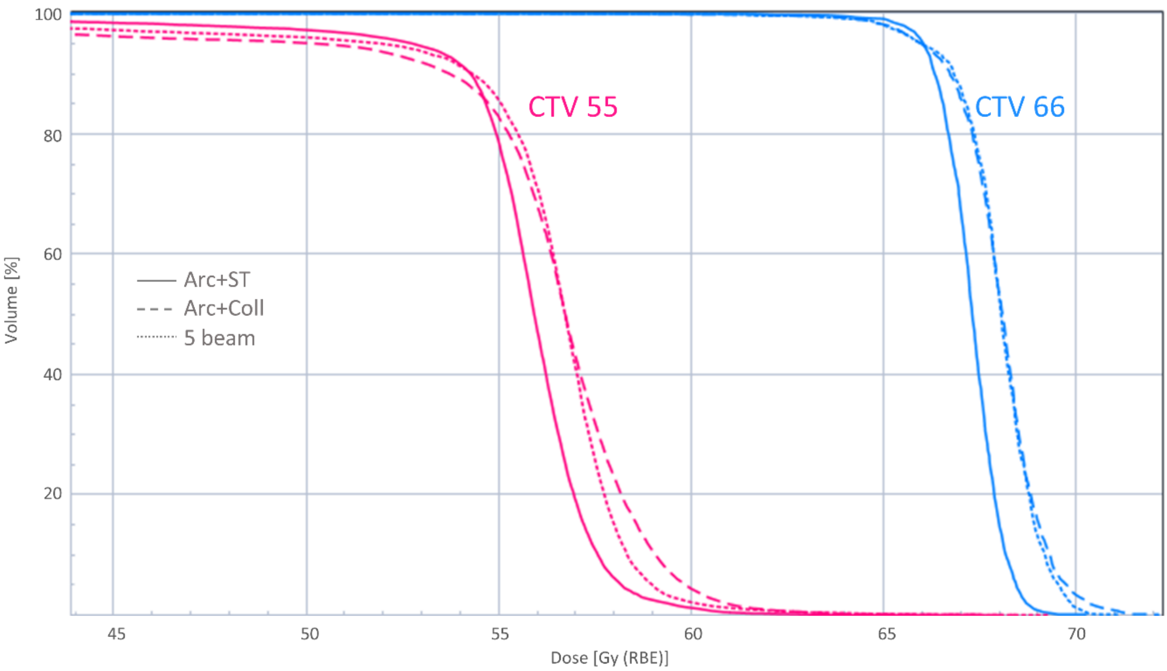

Figure S4. Enlargement of the DVHs in Figure 4 for the two targets CTV 55 (pink) and CTV 66 (blue) for Arc+ST (solid), Arc+Coll (dashed) and 5-beam (dotted) plans.

	Arc+ST	sub-Arc+ST	sub-Arc
No. EL	*360*	346	360
No. spots	*4281*	3797	3507
% ST	*44.84*	41.41	0
CI, CTV66	*0.48*	0.48	0.42
CI, CTV55	*0.36*	0.36	0.33
HI, CTV66	*0.96*	**0.97**	0.95
HI, CTV55	*0.91*	0.91	0.83
Worst-case, CTV66 D95 [%]	*99.2%*	99.2%	98.2%
Worst-case, CTV55 D95 [%]	*76.5%*	**77.1%**	70.3%
External, D_{mean} [Gy_{RBE}]	*6.4*	**6.2**	7.1
SpinalCord, D_{max} [Gy_{RBE}]	*20.1*	21.9	23.3
Parotid (L+R), D_{mean} [Gy_{RBE}]	*10.3*	10.2	10.6
Submandibular (L+R), D_{mean} [Gy_{RBE}]	10.7	10.7	11.6

Table S1. Complementary table to Table 2 with more plans for the H&N case. The Arc+ST plan is included also here as the reference. The sub-Arc plans are uncollimated with and without ST.
